# Lipid accelerating the fibril of islet amyloid polypeptide aggravated the pancreatic islet injury in vitro and in vivo

**DOI:** 10.1186/s12944-018-0694-8

**Published:** 2018-03-09

**Authors:** Xiao-Dan Mo, Li-Ping Gao, Qing-Jun Wang, Jie Yin, Yu-Hong Jing

**Affiliations:** 1 Institute of Anatomy and Histology & Embryology, Neuroscience, School of Basic Medical Sciences, Lanzhou University, No. 199 of Donggang West Road, Lanzhou City, Gansu Province 730000 People’s Republic of China; 2 Key Laboratory of Preclinical Study for New Drugs of Gansu province, Lanzhou University, No. 199 of Donggang West Road, Lanzhou City, Gansu Province 730000 People’s Republic of China; 3 Institute of Biochemistry and Molecular Biology, School of Basic Medical Sciences, Lanzhou University, No. 199 of Donggang West Road, Lanzhou City, Gansu Province 730000 People’s Republic of China

**Keywords:** IAPP, Lipid, Pancreatic islet, T2DM, Insulin, Palmitate

## Abstract

**Background:**

The fibrillation of islet amyloid polypeptide (IAPP) triggered the amyloid deposition, then enhanced the loss of the pancreatic islet mass. However, it is not clear what factor is the determinant in development of the fibril formation. The aim of this study is to investigate the effects of lipid on IAPP fibril and its injury on pancreatic islet.

**Methods:**

The fibril form of human IAPP (hIAPP) was tested using thioflavin-T fluorescence assay and transmission electron microscope technology after incubated with palmitate for 5 h at 25 °C. The cytotoxicity of fibril hIAPP was evaluated in INS-1 cells through analyzing the leakage of cell membrane and cell apoptosis. Type 2 diabetes mellitus (T2DM) animal model was induced with low dose streptozotocin combined the high-fat diet feeding for two months in rats. Plasma biochemistry parameters were measured before sacrificed. Pancreatic islet was isolated to evaluate their function.

**Results:**

The results showed that co-incubation of hIAPP and palmitate induced more fibril form. Fibril hIAPP induced cell lesions including cell membrane leakage and cell apoptosis accompanied insulin mRNA decrease in INS-1 cell lines. In vivo, Plasma glucose, triglyceride, rIAPP and insulin increased in T2DM rats compared with the control group. In addition, IAPP and insulin mRNA increased in pancreatic islet of T2DM rats. Furthermore, T2DM induced the reduction of insulin receptor expression and cleaved caspase-3 overexpression in pancreatic islet.

**Conclusions:**

Results in vivo and in vitro suggested that lipid and IAPP plays a synergistic effect on pancreatic islet cell damage, which implicated in enhancing the IAPP expression and accelerating the fibril formation of IAPP.

## Background

Type 2 diabetes (T2DM) is characterized by the hyperlipidemia and aberrant metabolism. Aberrant metabolism caused the serials of symptoms in diabetic patients including overweight or obesity, unbalance of anabolism and catabolism in adipocyte, chronic inflammation which induced the insulin resistance. Amyloid deposition consisted of islet amyloid polypeptide (IAPP) can be observed in pancreatic biopsy of patient with T2DM. IAPP is a 37-amino acid peptide of the calcitonin gene family. It is the most abundant component of pancreatic amyloid [1, 2]. IAPP has been suggested to be toxic to β-cells and to be involved in the development of T2DM [3, 4]. It has been proposed that overexpression of IAPP contributes to pancreatic amyloid formation and development of T2DM, this viewpoint supported by transgenic mouse and rat studies involving the overexpression of human IAPP (hIAPP) in islets of Langerhans [5]. Clinical studies indicate that hIAPP preferentially forms the amyloid deposition that implicates the pathology of islet hyalinization which is the pathological characteristic of T2DM [6–8]. Amyloid deposition is observed not only in patients with T2DM but also in some overweight or non-diabetic individuals [9–11]. Therefore, evidence suggests that some unidentified factors are involved in the regulation of IAPP secretion and amyloid deposition. Several studies have reported that the high expression of IAPP can be induced by lipid condition [4, 12–14]. It is known to cause the typical characteristic of T2DM [15, 16]. But how and what accelerating amyloid deposition by lipid is not clear.

Amyloid deposition is initiated by changed of protein conformation. Especially, the fibril form is preference to accumulate, which damage the cell membrane and induced the cell apoptosis [17–21]. The aim of the present study is to explore the effects of lipid on fibril formation of IAPP and the toxicity of fibril IAPP on pancreatic islet cells. The T2DM animal model was established by feeding with high fat diet or streptozotocin (STZ) injection, the IAPP levels and the pancreatic islet pathology and function were investigated. High dose STZ badly impairs the insulin secretion by inducing β-cell death mimicking type 1 diabetes. Low-dose STZ has been known to induce a mild impairment of insulin secretion which is similar to the feature of the later stage of type 2 diabetes [22, 23]. The investigators have been developed a rat model by feeding the animal with high-fat diet following low-dose STZ that closely mimic the metabolic characteristics of human type 2 diabetes [24].

## Methods

### Reagents

The hIAPP was synthesized using *t*-boc chemistry and purified by reverse phase high-performance liquid chromatography (Shanghai Zi Yu Biotech Co. Ltd., Shanghai, China). 1,1,1,3,3,3-Hexafluoro-2-propanol (HFIP), palmitate (PA), thioflavin-T (ThT) and STZ were purchased from Sigma (St. Louis, MO, USA). Enzymatic diagnostic kits for plasma glucose and triglyceride were purchased from Randox (Crumlin Co., Antrim, UK). The lactate dehydrogenase (LDH) assay kit and TUNEL kit were purchased from Roche (Roche, USA). The sandwich enzyme-linked immunosorbent assay (ELISA) kits of insulin and rIAPP were obtained from R&D (R&D, IL, USA). The anti-IAPP, and IRβ antibodies were purchased from Abcam (Cambridge, UK). Anti-Cleaved caspase3 antibody was purchased from Cell signaling (Cell signaling Technology, MA, USA). The anti-glyceraldehyde 3-phosphate dehydrogenase (GAPDH) antibody was obtained from Santa Cruz (Santa Cruz, CA, USA). The RPMI-1640 medium and the fetal bovine serum (FBS) were purchased from Gibco BRL (Gaithersburg, MD, USA). The water used in all experiments was ultrapure, and supplied by a Milli-Q water purification system from Millipore.

### ThT fluorescence assays

ThT based fluorescence assays were performed to evaluate the hIAPP fibril form. Briefly, hIAPP was first dissolved in HFIP and sonicated for 2 min to homogenize the sample. hIAPP was then diluted in 25 mM PBS (pH 7.4) containing 50 mM NaCl and 1% HFIP to a final concentration of 20 μM. The hIAPP were co-incubated with 200 μM PA at 25 °C for 5 h. Samples were aliquoted at designated time intervals and ThT based fluorescence assays were used to detect the fibril formation of hIAPP; the fluorescence emission experiments were performed on a Hitachi FL-2700 fluorometer (Toyko, Japan) with the excitation and emission wavelengths set at 450 and 482 nm, respectively. All experiments were repeated for at least three times.

### Transmission electron microscopy (TEM)

20 μM hIAPP were co-incubated with 200 μM PA at 25 °C for 5 h, then Five microliters of samples to be imaged were spotted on a 300 mesh Formvar-carbon coated copper grid (Shanghai, China) and stained with 1% freshly prepared uranyl formate. Samples were air dried and observed under a JEM2100 TEM (JEMO, Toyko, Japan) operating at an accelerating voltage of 100 kV.

### Cell culture and treatment

The rat insulinoma cell line (INS-1) was purchased from the Institute of Biochemistry and Cell Biology of Shanghai. The cells were briefly maintained in an RPMI-1640 medium supplemented with 10% FBS, 2 mmol/L L-glutamine, 1 mmol/L sodium pyruvate, 10 mmol/L HEPES, 50 mmol/L mercaptoethanol, 100 U/mL penicillin, and 100 U/mL streptomycin at 37 °C in a humid atmosphere (95% relative humidity, 5% CO2). For the peptide treatment, lyophilized hIAPP was dissolved in HFIP, which was removed by evaporation under N2. The hIAPP incubation with palmitate for 5 h at 25 °C, then used to treat cells. INS-1 cells were incubated with 20 μM and 50 μM fibril hIAPP for 24 h to evaluate the LDH leakage, levels of insulin mRNA and apoptosis.

### LDH assay

The LDH release indicates the change of membrane permeability, which can reflect the damage extent of the cell membrane. The cells were precipitated by centrifugation (1500×g) for 10 min at room temperature at the end of the treatments. The supernatants were transferred to a 96-well plate. The LDH activity was assayed using the cytotoxicity detection kit according to the manufacturer’s instructions. The cells treated with 1% Triton X-100 were used as high control, while media without cells served as a low control. The results were expressed as % LDH leakage [(experimental value − low control) / (high control − low control) × 100] [25].

### Animals and treatment

A total of 48 healthy male Sprague Dawley (SD) rats weighing 200-220 g were purchased from the Experimental Animal Center of Lanzhou University. The animals were housed in a standard environment at 20–25 °C and 50–70% humidity and maintained under a 12 h light–dark cycle with food and water ad libitum. All animal experimental protocols were approved by the institutional Animal Ethics Committee, Lanzhou University (permission number: SCXK Gan 2009–0004). The animals were randomly divided into four groups as follows: control group (single injection with equal volume of 0.1 M citrate buffer through peritonea, feeding with regular diet), STZ group (single injection with STZ through peritonea, 30 mg/kg, STZ dissolved in 0.1 M citrate buffer, feeding with regular diet), HD group (single injection with equal volume of 0.1 M citrate buffer through peritonea, feeding with high-fat diet), and STZ + HD group (single injection with STZ through peritonea, 30 mg/kg, STZ dissolved in 0.1 M citrate buffer, feeding with regular diet). Diets (Ke Ao Co. Ltd., Beijing) contained 15% (low fat) or 36% (high fat) calories derived from fat. Fat was provided as corn oil and hydrogenated coconut oil, with the ratio of saturated to unsaturated fatty acids being 1:3 in each diet. The progressively increasing amounts of fat were balanced by decreasing amounts of carbohydrate (65 kcal % and 44 kcal % in low- and high-fat diets, respectively) and constant amounts of protein (20 kcal%).

### Examination of plasma rIAPP, insulin, triglyceride and glucose in diabetic rats

Plasma was isolated by low-speed centrifugation of blood at 4 °C. The plasma glucose and triglyceride were measured with respective enzymatic diagnostic kits according to the manual instruction. Plasma insulin and rIAPP were measured by respective ELISA kit according to the manual instruction. All data were obtained from two independent measurements, each with triplicate incubations.

### Glucose tolerance test (GTT) and insulin sensitivity tests (IST)

For the glucose tolerance and insulin sensitivity tests, the rats were intraperitoneally injected with glucose (2 g/kg) or subcutaneously injected with human regular insulin (0.75 unit/ kg) after 12 h of fasting, respectively. Blood samples were collected from the tail vein at 0 min, 30 min, 60 min, 90 min, and 120 min after treatment. The blood glucose levels were measured.

### Histology examination of pancreatic islet

After two months, four rats, which were randomly selected from each group, were anesthetized. The pancreatic tail was isolated (in a size of 0.5 × 0.5 × 0.5 cm) and performed on formalin-fixed. The paraffin-embedded pancreas specimens were cut at a 5 μm thickness. Sections were collected and stained by hematoxylin-Eosin (H-E) to evaluate the histopathological changes of pancreatic islet. To identify the pancreatic islet, sections were stained with insulin antibody (1:100) overnight at 4 °C. Then the sections were rinsed with 0.01 M PBS and incubated with FITC-IgG (1:50). The sections were observed under fluorescence microscope and scanned in dark field. The other sections were used to incubate using the rIAPP primary antibody (1:100), overnight at 4 °C. The sections were rinsed with 0.01 M PBS and incubated with the corresponding second antibodies at 37 °C for 1 h, then rinsed and incubated with streptavidin-conjugated horseradish peroxidase (1:100) at 37 °C for 1 h. The immunoreactivity was visualized with 0.05% a diaminobenzidine (DAB) as a chromogen. The sections were observed under a microscope and scanned in bright field. The pancreatic islets were manually outlined. The pancreatic islet area was calculated using ImageJ software. The optical density was used to quantify the rIAPP expressed by normalized with unit area of pancreatic islet.

### TUNEL assay

The paraffin-embedded pancreas specimens were cut at a 5 μm thickness. And terminal deoxynucleotidyl transferase-mediated deoxyuridine triphosphate biotin nick end labeling (TUNEL) assay was performed on the sections. Sections were treated with 0.2% H_2_O_2_ for 10 min, rinsed in 0.1 M PBS, and incubated with Proteinase K Solution (contain 200 mmol Tris, pH, 7.4, 0.5 mmol EDTA, and proteinase K 1 mg/ml) for 20 min at room temperature. Then the sections were incubated in TUNEL reaction mixture using the In Situ Cell Death Detection Kit for 1 h at 37 °C, then rinsed in 0.1 M PBS three times for 5 min and incubated in Converter peroxidase (POD) for 30 min at 37 °C, rinsed in 0.1 M PBS three times for 5 min, and color-developed with SIGMA FAST, a DAB POD substrate. The pancreatic islets were manually outlined. The TUNEL positive cells were counted and normalized with unit area of pancreatic islet.

### Procedure of pancreatic islet isolation

After two months, eight rats, which were randomly selected from each group, were anesthetized. And the pancreas was isolated. After enzymatic digestion of the pancreas, islets of Langerhans were purified using a discontinuous density gradient of ficoll solutions as previously reported [26], with minor adjustments. Briefly, the common bile duct was cannulated and 1 ml of digestion solution (0.2 mg/ml collagenase in HBSS containing 10 mmol HEPES) was injected to distend the pancreas. The pancreas was removed and placed for 15 min at 37 °C in a Petri dish filled with 5 ml of digestion solution to isolate the islet.

### RNA extraction and Q-RT-PCR

The total RNA was extracted from the isolated pancreatic islet or INS-1 cells using the RNAiso plus reagent (Takara Biotech, Co., Ltd., Dalian, China) and depleted of contaminating DNA with RNase-free DNase according to the manufacturers’ instructions. cDNA was synthesized from 1 μg of RNA with M-MuLV reverse transcriptase and random hexamer according to the manufacturer’s instructions (Fermentas, Burlington, Canada). Q-RT-PCR was performed using the PIKoREAl96 detector (Thermo Scientific, USA). The primers for the rIAPP were 5′-GCCCACTGAAAGGGATCTTG-3 (forward) and 5′-GCACTTCCGTTTGTCCACCT-3′ (reverse). The primers for the rat preproinsulin were: 5′-CAGCACCTTTGTGGTTCTCACTT-3′ (forward) and 5′-CTCCACCCAGCTCCAGTTGT-3′ (reverse). The rat GAPDH primers were 5′-GGCACAGTCAAGGCTGAGAATG-3′ (forward) and 5′-ATGGTGGTGAAGACGCCAGTA-3′ (reverse). The assays were initiated for 5 min at 95 °C and 40 cycles of 15 s at 94 °C and 1 min at 60 °C. The relative levels of amylin or preproinsulin mRNA expression were calculated using the 2^ΔCT^ method.

### Protein extraction and western blot analysis

The total proteins were extracted from pancreatic islet or INS-1 cells using RIPA buffer that contains protease inhibitors. The proteins (50 μg) were fractionated on 10% sodium dodecyl sulfate polyacrylamide gel electrophoresis, and then transferred into polyvinylidene fluoride membranes. The membranes were blotted with anti-IRβ (1:1000), anti-caspase-3 (1:1000), and anti-GAPDH (1:5000) antibodies, as well as with horseradish peroxidase-conjugated second antibody (1:5000). The immunoreactive protein bands were visualized by enhanced chemiluminescence.

### Statistical analysis

The data were expressed as mean ± SEM. Statistical analysis was performed using SPSS statistical program at 17.0version. The difference between two groups was analyzed by Student’s *t*-test, whereas that among three or more groups was analyzed by one-way or two way analysis of variance with least significant difference test. The difference with *P* < 0.05 was considered statistically significant.

## Results

### Effects of lipid on fibrillation of hIAPP

**Fig. 1:**
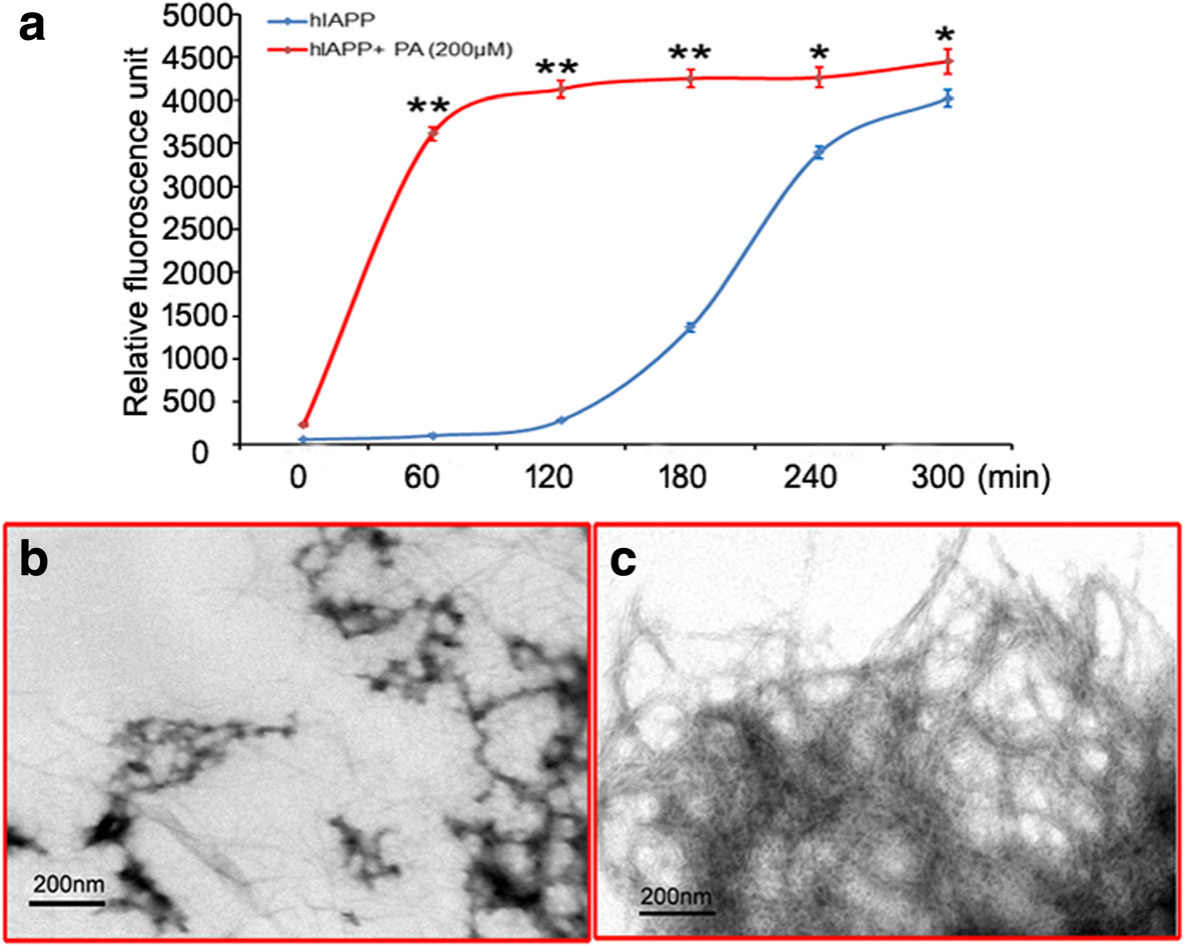
Effects of palmitate on the fibrillation of hIAPP. **a** Relative thioflavin-T fluorescence intensity. **b** TEM image of hIAPP alone. **c** TEM image of hIAPP co-incubated with palmitate. * denotes *p* < 0.05 and ** denotes *P* < 0.01 compared to the hIAPP. *n* = 3. One-way ANOVA was used with post hoc test

Twenty μM hIAPP incubated with 200 μM palmitate at 25 °C, and then ThT-based fluorescence assays were performed. hIAPP exhibited maximum ThT emission with a long lag time of 4.3 ± 0.6 h (Fig. 1a) compared with palmitate treated-hIAPP (1.5 ± 0.2 h, Fig. 1a). This result suggested palmitate accelerated the formation of fibril hIAPP. Under TEM, a mesh of typical long linear fibrils was detected for palmitate treated-hIAPP incubated for 5 h (Fig. 1c). In contrast, only a few linear fibrils together with significant amount of amorphous aggregates were observed in untreated hIAPP (Fig. 1b), which is consistent with the results by ThT assay.

### Effects of fibril hIAPP on membrane permeability of pancreatic cells

**Fig. 2:**
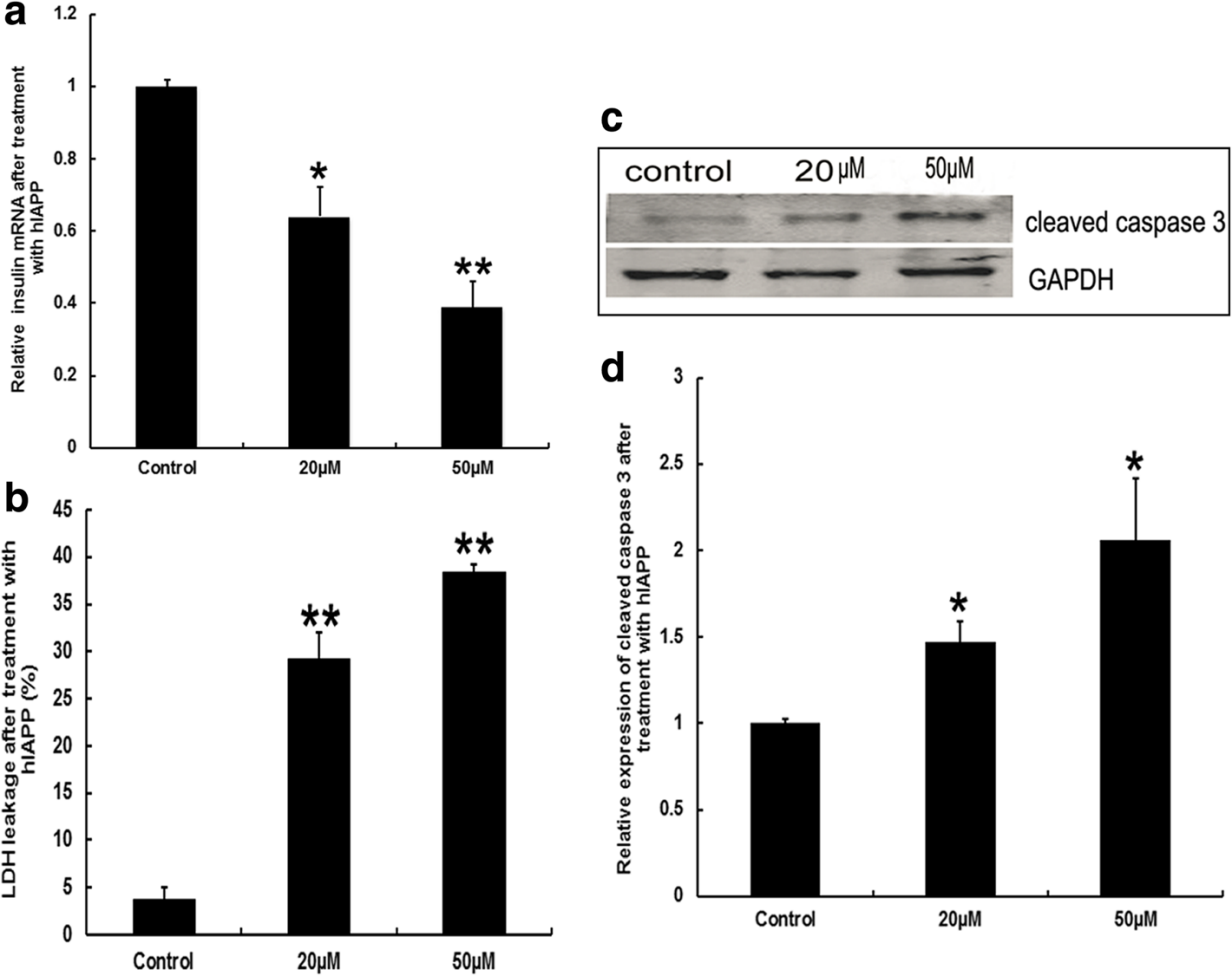
Effects of fibril hIAPP on INS-1 cells. **a** mRNA levels of insulin. **b** Membrane permeability evaluated by LDH leakage assay. **c** Representative images of WB for cleaved aspase-3 expression. **d** Relative quantitation of cleaved caspase-3 expression was performed. * denotes *p* < 0.05 and ** denotes *P* < 0.01 compared to the control group. n = 3. One-way ANOVA was used with post hoc test

The INS-1 cell, which was the cell line of insulinoma characterized by insulin secretion were incubated with exogenous fibril hIAPP for 24 h at concentrations of 20 μM and 50 μM to further confirm the direct effect on the INS-1 cells. The results showed that the insulin mRNA levels decreased about 50% after treatment with fibril hIAPP (Fig. 2a). These findings also suggested that fibril hIAPP may repress the transcription of insulin gene. Membrane permeability was evaluated with the ratio of the LDH leakage. As shown in Fig. 2b, the LDH leakage increased to 6–8 folds after incubation with fibril hIAPP. This result suggested that hIAPP aggravated the cell damage in a dose-dependent manner. In addition, the hIAPP treatment induced the caspase-3 high expression, which indicated the initiation of DNA damage and cell apoptosis (Fig. 2c and d).

### Changes of body weight, GTT and IST in diabetic rats

**Fig. 3:**
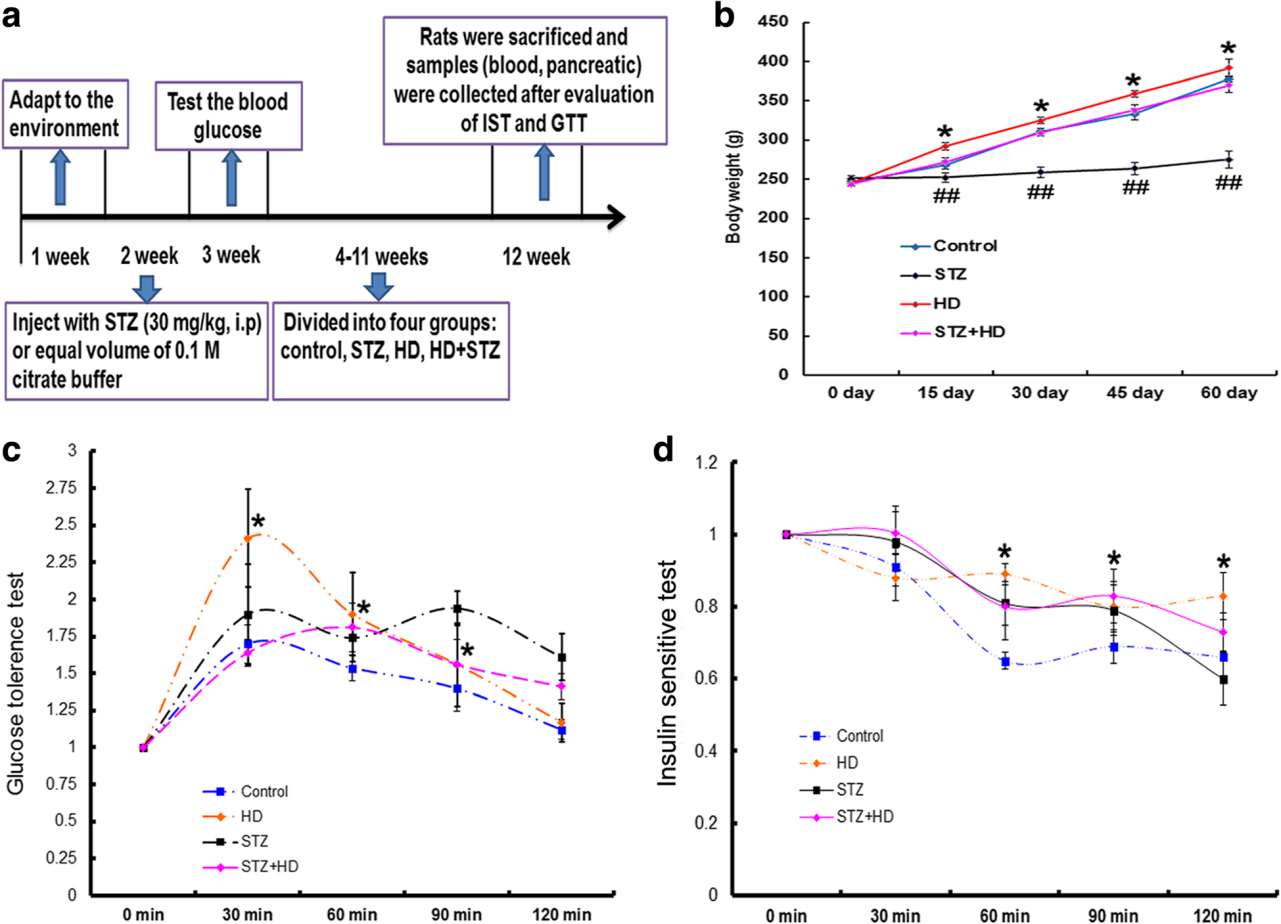
Body weight, fast glucose tolerance and insulin sensitivity after treating with high-fat diet, STZ injection, and STZ injection combined with high-fat diet. **a** Flow chart of animal experiment. **b** The extent of body weight gain duration of two months. * denotes *p* < 0.05 in the HD group vs. control group; ## denotes *p* < 0.01 in the STZ group vs. control group. *n* = 12. **c** Glucose tolerance is evaluated after 12 h fasting. * denotes *p* < 0.05 in the HD group vs. control group, n = 12. **d** Insulin sensitivity was evaluated after 12 h fasting, * denotes *p* < 0.05 in the HD group vs. control group. n = 12. Two-way ANOVA was used with post hoc test

As shown in Fig. 3b, the body weight gain in high-fat diet group is more than in the control. Alternatively, body weight gain in STZ treated group is less than in the control. And the extent of body weight gain in STZ + HD group is similar as the control. The glucose tolerance increased about one fold and the insulin sensitivity decreased about 20% in the HD and HD + STZ groups, respectively, compared with that in the control group (Fig. 3c and d).

### Changes of plasma rIAPP, insulin, triglyceride and glucose in diabetic rats

**Fig. 4:**
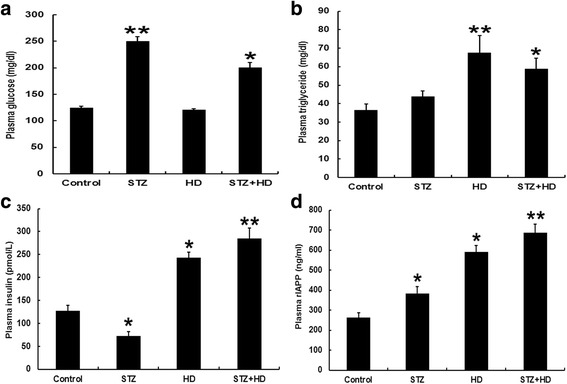
Blood biochemical parameters of rats after treating with high-fat diet, STZ injection, and STZ injection combined with high-fat diet for 2 months. **a** Plasma glucose. **b** Plasma triglyceride. **c** Plasma insulin. **d** Plasma rIAPP. * denotes *p* < 0.05 compared with the control group; ** denotes *p* < 0.01 compared with the control group. n = 12. Two-way ANOVA was used with post hoc test

As shown in Fig. 4a, Plasma glucose was higher in the STZ (about 250 mg/dl) and STZ + HD (about 220 mg/dl) groups than that in the control group after two months of treatment. Among them, plasma glucose increased significantly in the STZ group. Plasma triglyceride was higher in the HD (about 70 mg/dl) and STZ + HD (about 60 mg/dl) groups than that in the control group. Among them, plasma triglyceride significantly increased in the HD group (Fig. 4b). The plasma insulin decreased in STZ group, and increased in HD and HD + STZ group compared with the control group (Fig. 4c). Plasma rIAPP significantly increased in HD + STZ group compared with the control group (*p* < 0.01, Fig. 4d). Also, rIAPP increased in STZ and HD groups compared with the control group (*p* < 0.05, Fig. 4d).

### Changes of pancreatic islet pathology in diabetic rats

**Fig. 5:**
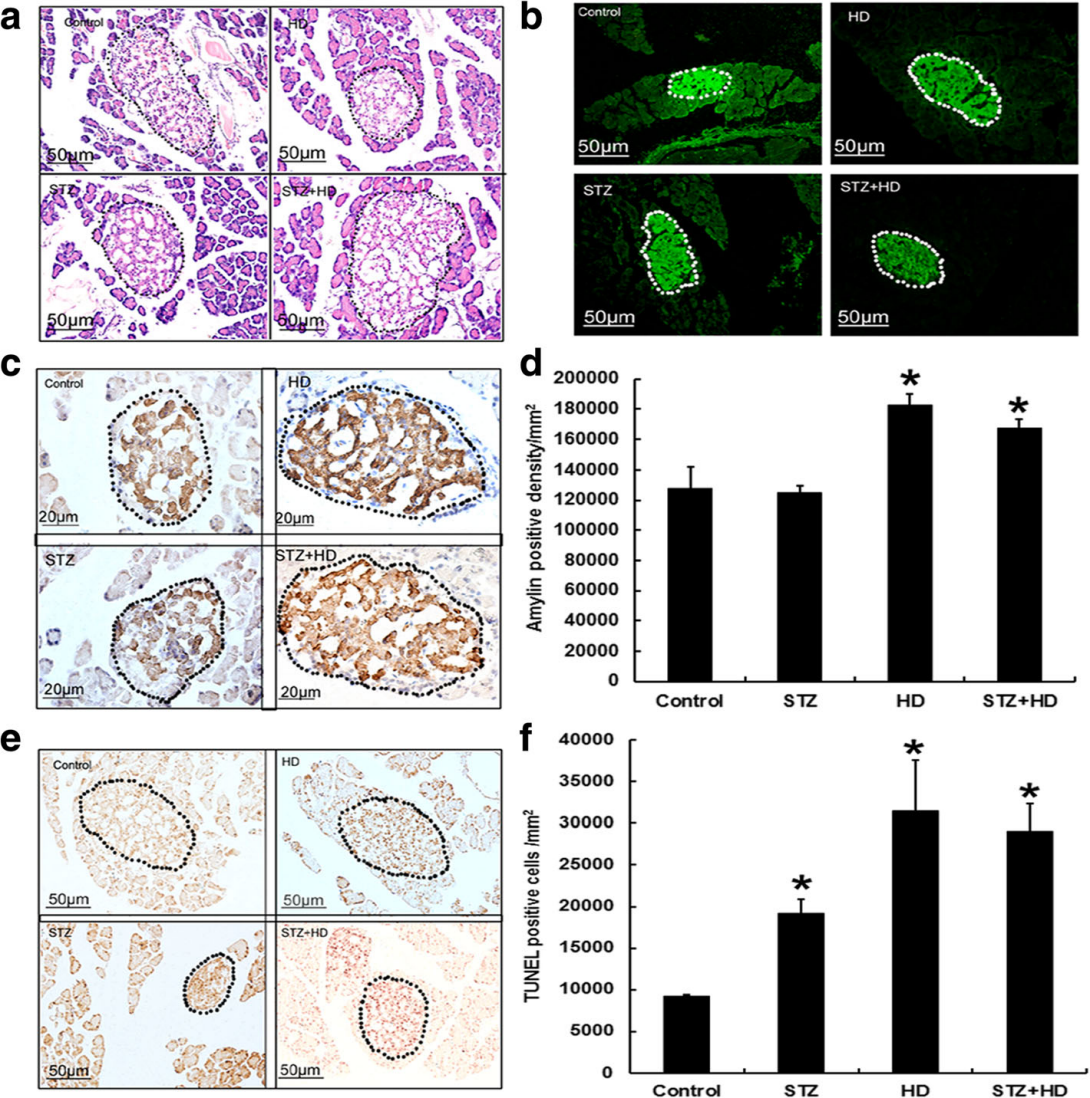
Morphology of pancreatic islet after treatment with STZ injection, high-fat diet, and STZ injection combined with high-fat diet in rats. **a** Representative images of H-E staining to show the islet by dashed lines. **b** Representative images of insulin immunoflurorescence staining to show the islet by dashed lines. **c** Representative images of amylin expression in the islet (outlined by dashed lines). **d** Levels of amylin expression calculated with ImageJ software in sixteen islets from four rats each group. **e** Representative images of TUNEL staining in the islet (outlined by dashed lines). **f** Numbers of TUNEL positive cells in the islet were calculated and normalized by unit area of pancreatic islet. * denotes *p* < 0.05 compared with the control group. *n* = 4. Two-way ANOVA was used with post hoc test

To confirm the pancreatic islet morphology and exclude the inflammation which induced by STZ injection, we observed the pancreatic islet by H-E staining under microscope. As shown in Fig. 5a, the boundary is obvious between islet and acinus. We did not find inflammatory cell infiltration in islet. To prove islet function, the insulin expression in islet was detected by immunofluorescence. Results showed insulin immunoreaction is positive in islet from control, HD, STZ and STZ + HD groups (Fig. 5b). The rIAPP in the islet were evaluated using immunohistochemistry and semi-quantitative methods. As shown in Fig. 5c and d, the rIAPP expression increased in the islet of the HD and HD + STZ groups compared with the control group. No significant difference was found in the STZ group compared with the control group. To evaluate the cell apoptosis, we detected the number of TUNEL positive cells in islet. As shown in Fig. 5e and f, numbers of TUNEL positive cell increased in the HD, STZ, and HD + STZ groups compared with the control group.

### Effects of HD on isolated pancreatic islet

**Fig. 6:**
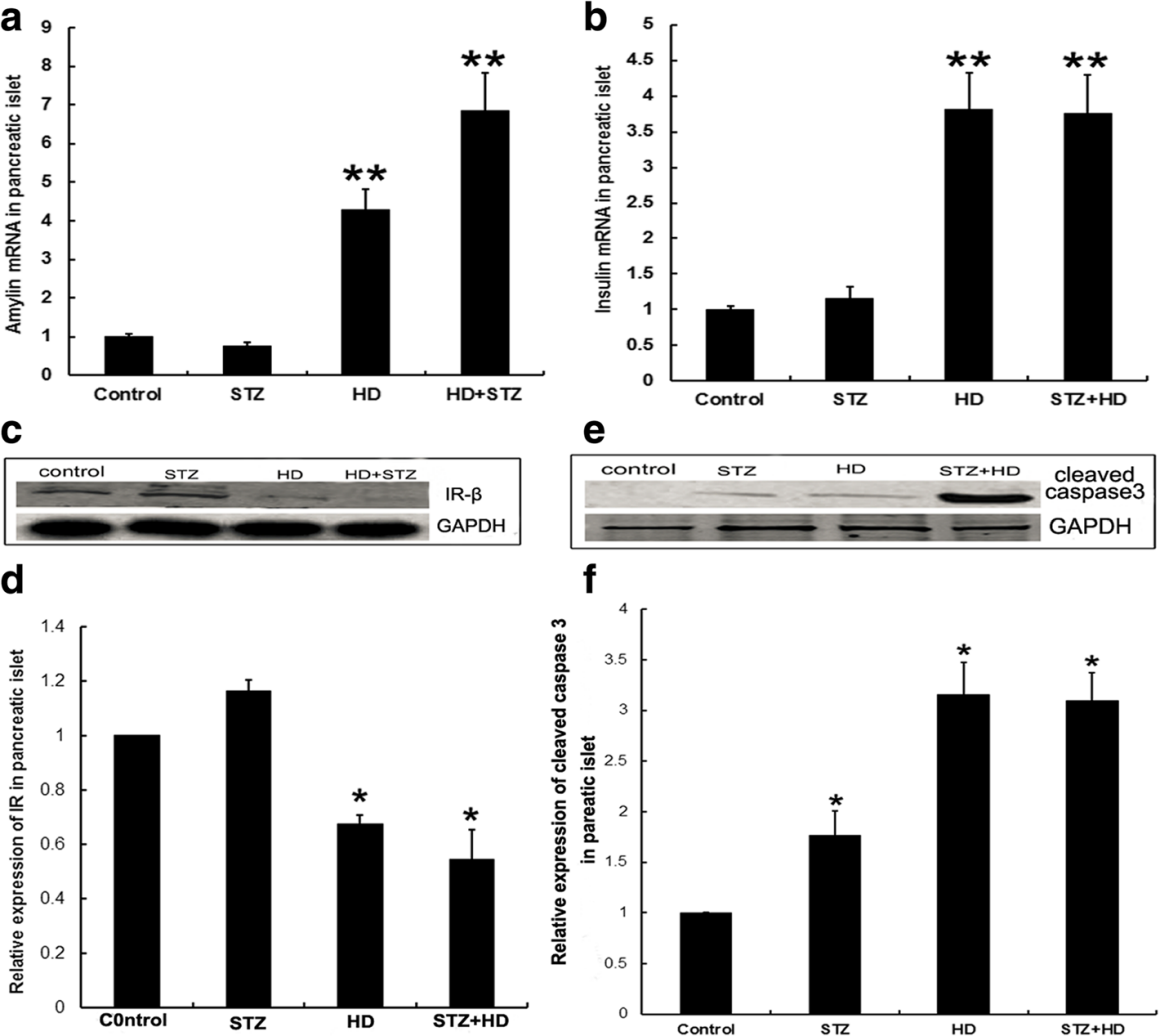
Isolated pancreatic islet function was evaluated after treatment with STZ injection, high-fat diet, and STZ injection combined with high-fat diet in rats. **a** mRNA levels of amylin. **b** mRNA levels of insulin. **c** Representative images of WB for IRβ expression. Relative quantitation of IRβ expression was performed and shown in (**d**). **e** Representative images of WB for cleaved caspase-3 expression. **f** Relative quantitation of cleaved caspase-3 expression was performed. * denotes *p* < 0.05 compared with the control group; ** denotes *p* < 0.01 compared with the control group. n = 4. Two-way ANOVA was used with post hoc test

To quantify the rIAPP expression in pancreatic islet, the islet was isolated from pancreas. The rIAPP mRNA increased in HD and STZ + HD groups compared with the control, but no difference in STZ group (Fig. 6a). Similarly, the insulin mRNA in HD and STZ + HD groups, but not STZ group increased compared with the control (Fig. 6b). Total protein was extracted from isolated pancreatic islet, and the levels of IR-β and caspase-3 were measured by western blot. The results showed IR-β expression decreased in HD and STZ + HD groups compared with the control (Fig. 6c and d). Additionally, the caspase-3 increased in STZ, HD and STZ + HD groups compared with the control (Fig. 6e and f).

## Discussion

Amyloid deposition plays a critical role in many different human diseases, including Huntington’s disease, Parkinson’s disease (PD), Alzheimer’s disease (AD) and T2DM. Among these diseases, T2DM together with AD are leading causes of morbidity and mortality in the elderly. Both diseases share common clinical and biochemical features [27], including functional tissue loss due to accumulation and aggregation of small peptides, such as IAPP in the pancreas of T2DM patients, or beta amyloid in AD patients. Specifically, IAPP fibrils arise following initial increased production of IAPP which leads to oligomeric aggregation of IAPP molecules that then assemble into amyloid fibrils in pancreatic islets, eventually resulting in pancreatic beta cell loss [28–30]. More secretion of IAPP is the precondition, but not the determinant factor in amyloid deposition. Increasing literatures have reported IAPP fibrils play the dramatic roles in the development of amyloid deposition. Therefore, facilitation of fibril formation accelerates the amyloid deposition. In our present study, we found incubation of hIAPP with palmitate increased the fibril form which caused the increase of membrane permeability and apoptosis in INS-1 cells. In type 2 diabetic rats induced by feeding with high fat diet, we fund rIAPP expression increased accompanied pancreatic islet cell apoptosis, but no typical amyloid deposition. In isolated pancreatic islet tissue, we fund rIAPP mRNA and insulin mRNA increased, but insulin receptor decreased in diabetic rats, which may implicate the abnormal insulin signals. Given the insulin signals play pivotal roles in cell survival, pancreatic cell apoptosis partially contributed to the reduction of insulin signaling.

IAPP is co-localized with insulin in the islet beta-cells and is co-secreted with insulin in response to beta-cell stimulation by both glucose and non-glucose secretagogues agents, such as arginine [31]. Therefore, therapies that alter endogenous insulin secretion are likely to cause parallel changes in IAPP secretion. Both insulin and IAPP gene expression and release by pancreatic islets are regulated by glucose [13, 32–34]. In vitro, the study showed that palmitate and oleate cause transcriptional induction of amylin gene in beta-cells and murine islets. This induction is mediated by the Ca^2+^-PKC signaling pathway and de novo synthesized proteins. However, the effect of lipid on the expression and release of IAPP by pancreatic islet in vivo is not clear. Our present studies indicate high fat diet combined with the low dose STZ injection induced the features of T2DM characterized by decrease of insulin sensitivity and increase of plasma insulin, glucose and triglyceride, as well plasma rIAPP augment and islet cell apoptosis. To confirm the changes of pancreatic islet under high fat diet consumption, pancreatic islet was isolated and its function was analyzed. These results suggested the transcription of insulin and amylin were affected under high fat diet condition. Together with all data in vivo, we think simultaneous changes of IAPP expression, glucolipid metabolism and insulin sensitivity caused islet cell apoptosis which contributed to the onset of T2DM.

The most widely accepted hypothesis is that IAPP-induced cytotoxicity occurs via a membrane disruption mechanism. The experimental evidence suggested that the peptide A*β*, involved in Alzheimer’s disease, could form cation-selective channels in planar lipid bilayers [13]. Similar experiments showed that hIAPP could also form cation-selective channels and ultimately disrupt the membranes [35]. These channels have been also observed for other amyloidogenic proteins suggesting that the toxicity of amyloid proteins seems to be linked to their shared potential to form pores in membrane [36, 37]. Many studies suggest hIAPP could induce membrane damage. But the exact mechanism of hIAPP-induced membrane disruption is far from clear. And numerous models have been described during recent years [38–41]. A report concluded that soluble oligomers from several types of amyloids, including hIAPP, specifically increase lipid bilayer conductance, while the soluble low molecular weight species have no effect, suggesting that this may represent the common primary mechanism of pathogenesis in amyloid-related diseases, such as AD, PD and diabetes [42].

The lipid condition accelerated the IAPP toxicity not only based on its high expression but also on the associated IAPP conformation. The dynamics and extent of IAPP oligomerization and aggregation were shown to be important parameters of IAPP toxicity [40, 43, 44]. However, the absence of the islet amyloid in several transgenic mouse strains with very high amylin production contradicted this suggestion. Therefore, additional factors need to be considered. The islet amyloid occurred after a persistent intake of a high-fat diet in transgenic mouse models [42, 45]. Finding the effects of lipid on IAPP toxicity was a critical issue. A previous study suggested that free fatty acids (FFAs) can act as direct potent stimulators of amylin fibrillogenesis [46]. Our study showed that insulin resistance and rIAPP production occurred in rats feeding with high-fat diet. These led to a chronic overproduction of rIAPP. Another study indicated that FFAs not only enhanced fibril formation from synthetic hIAPP in vitro, but also rapidly led to the appearance of an abnormal intragranular material in cells of cultivated hIAPP transgenic mouse islets [47–49].

Cell damage with IAPP was caused by the increase of membrane permeability. Accordingly, evidence showed that various sizes of the hIAPP-induced membrane pores or openings, ranging from Ca^2+^-permeable to permeable for fluorescent dyes with a size larger than 1 kDa [50–52]. Soluble hIAPP and amyloid oligomers generally could have characteristics of pore-forming protein toxins like α-hemolysin, and might have a similar mechanism of action [53–55]. Our results also confirmed that cell membrane permeability increased after incubation with hIAPP in a dose-dependent manner.

## Conclusions

Fibril hIAPP directly damaged the pancreatic cells characterized by increase of permeability of cell membrane, inhibition of insulin secretion, and triggering cell apoptosis. Furthermore, high fat diet induced endogenous rIAPP secretion accompanied the insulin secretion, but in isolated pancreatic islet, high fat diet reduced the insulin receptor expression, suggesting the insulin signaling is inhibited. Insulin resistance and IAPP expression induced by fatty acids aggravated the pancreatic islet cell apoptosis in vivo. Together with evidence from in vivo and in vitro, suggested fatty acid and amylin plays the synergistic effect on pancreatic islet cell damage which involved in amylin high expression and fibril formation of IAPP, cell membrane leakage and cell apoptosis. Also, the limitation and deficiency in our experiment is non-blinded methods, which may produce the bias.

## Abbreviations

aa: amino acid
DAB: Diaminobenzidine
FAs: Fatty acids
FBS: Fetal bovine serum
GAPDH: Glyceraldehyde 3-phosphate dehydrogenase
HD: High-fat diet
HFIP: 1,1,1,3,3,3-Hexafluoro-2-propanol
hIAPP: human islet amyloid polypeptide
LDH: The lactate dehydrogenase
MTT: thiazolyl blue tetrazolium bromide
PA: Palmitate
Q-RT-PCR: Quantitative real-time PCR
rIAPP: rodent islet amyloid polypeptide
STZ: Streptozotocin
T2DM: Type 2 diabetes mellitus
TEM: Transmission Electronic Microscopy
ThT: thioflavin-T
